# The impact of body compositions on contrast medium enhancement in chest CT: a randomised controlled trial

**DOI:** 10.1259/bjro.20230054

**Published:** 2023-10-18

**Authors:** Mette Karen Henning, Trond Mogens Aaløkken, Anne Catrine Martinsen, Safora Johansen

**Affiliations:** 1 Faculty of Health Sciences, Department of Life Sciences and Health, Oslo Metropolitan University, Oslo, Norway; 2 Department of Radiology and Nuclear Medicine, Oslo University Hospital, Oslo, Norway; 3 Faculty of Medicine, University of Oslo, Oslo, Norway; 4 The Research Department, Sunaas Rehabilitation Hospital, Nesodden, Norway; 5 Department of Cancer Treatment, Oslo University Hospital, Oslo, Norway

## Abstract

**Objective:**

To compare a fixed-volume contrast medium (CM) protocol with a combined total body weight (TBW) and body composition-tailored protocol in chest CT.

**Methods and materials:**

Patients referred for routine contrast enhanced chest CT were prospectively categorised as normal, muscular or overweight. Patients were accordingly randomised into two groups; Group 1 received a fixed CM protocol. Group 2 received CM volume according to a body composition-tailored protocol. Objective image quality comparisons between protocols and body compositions were performed. Differences between groups and correlation were analysed using *t*-test and Pearson’s *r*.

**Results:**

A total of 179 patients were included: 87 in Group 1 (mean age, 51 ± 17 years); and 92 in Group 2 (mean age, 52 ± 17 years). Compared to Group 2, Group 1 showed lower vascular attenuation in muscular (mean 346 Hounsfield unit (HU) *vs* 396 HU; *p* = 0.004) and overweight categories (mean 342 HU *vs* 367 HU; *p* = 0.12), while normal category patients showed increased attenuation (385 *vs* 367; *p* = 0.61). In Group 1, strongest correlation was found between attenuation and TBW in muscular (*r* = −.49, *p* = 0.009) and waist circumference in overweight patients (*r* = −.50, *p* = 0.005). In Group 2, no significant correlations were found for the same body size parameters. In Group 1, 13% of the overweight patients was below 250 HU (*p* = 0.053).

**Conclusion:**

A combined TBW and body composition-tailored CM protocol in chest CT resulted in more homogenous enhancement and fewer outliers compared to a fixed-volume protocol.

**Advances in knowledge:**

This is, to our knowledge, the first study to investigate the impact of various body compositions on contrast medium enhancement in chest CT.

## Introduction

In chest CT, contrast medium (CM) is required to assess, delineate, and differentiate between a wide range of thoracic disorders. Factors affecting CM enhancement include injection parameters; CM volume, injection rate and delay; and patient-related factors such as cardiac output and body size.^1^

CM volume in chest CT is most commonly fixed or adjusted to patient total body weight (TBW).^2^ A fixed-volume protocol may be effective for average-sized patients.^1^ However, patient populations are not homogenous. Therefore, this approach may lead to CM over- or underdosage resulting in non-uniform enhancement.^3–5^ One way of solving this is to relate CM volume linearly to TBW. This may reduce interpatient variability,^6–8^ but does not adapt CM volume to patient body composition. Because of the increased vascularisation in muscle tissue compared to fatty tissue, the blood volume does not increase linearly to TBW.^1,9–11^ Clinically, this discrepancy in blood volume may affect CM enhancement, resulting in insufficient enhancement in younger and muscular patient, while patients with higher fat mass, may receive unnecessarily high CM volumes.^3^ Therefore, several strategies to personalise CM volume have been suggested in the literature, none are, however, reported as superior.

Due to practical considerations, simplified CM volume adaptions can be utilised in daily clinical practice.^6,7,12^ One such strategy is the use of modified weight-based look-up tables.^13,14^ This patient-tailored approach is an easy applicable method to estimate a personalised CM volume in a clinical context, facilitating adaption of CM volume to body size or allometric parameters.^15^ However, this may introduce inaccuracy compared to methods utilising more individualised size predictors such as lean body weight or body surface area.^10,15^

The performance of various body weight adapted CM protocols have been explored for coronary arteries in CT angiography, and for abdominal CT.^4,7,9,16,17^ However, there is a lack of recognised guidelines and consensus related to CM administration in chest CT.^2^ Given the increasing prevalence of overweight and obesity in all segments of the population,^18^ it is important to identify the relationship between pharmacokinetic parameters such as CM distribution and body size. This identification may allow development of CM protocols suitable for patients of any size.

In this study, CM volumes are stratified across a range of body parameters to explore and provide knowledge related to CM administration for routine chest CT. The aim was to compare the performance of a fixed-volume CM protocol with a combined weight and body composition-tailored CM protocol in chest CT.

## Material and methods

### Ethics

This prospective study was approved by the Regional Committee for Medical and Health Research. All patients participated upon written and oral consent.

### Study population

**Figure 1.**
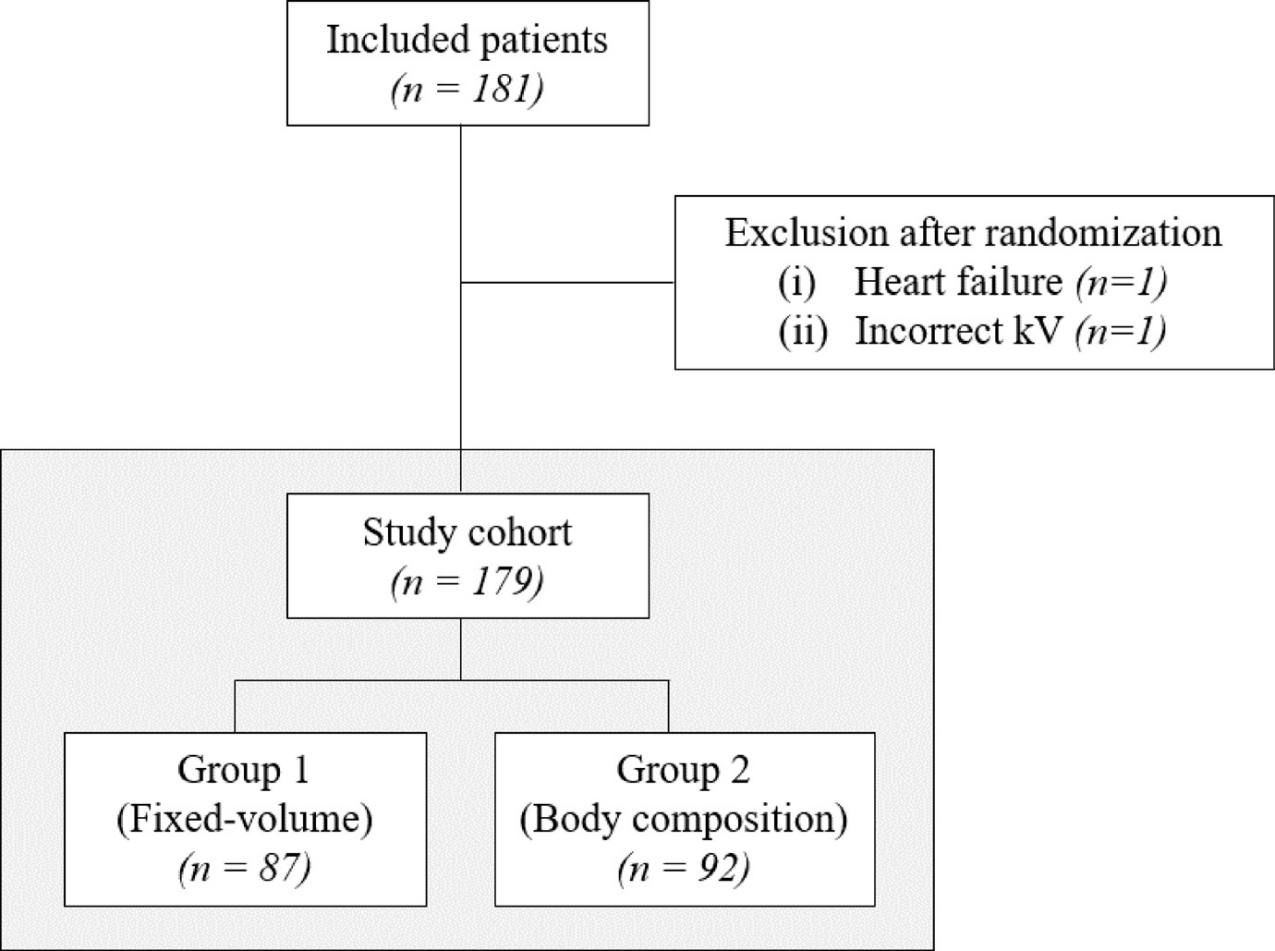
Flowchart of patient inclusion.

179 eligible patients undergoing chest CT examination between August 2019 and September 2021 at Oslo University Hospital were included (Figure 1). Exclusion criteria were haemodynamic instability, cardiac failure, pacemaker, renal insufficiency (estimated glomerular filtration rate <30 ml/min/1.73 m^2^), contraindications to contrast-enhanced CT, and age <18 years. Age, gender, TBW, height, body mass index (BMI), waist circumference, CM volume, and injection rate were recorded (Table 1). Some subjects (*n* = 89) from Group 2 have been included in a study estimating the variation in CM dose using various lean body mass methods,^19^ but all results are new for this study.

**Table 1.** Patient characteristics, CM parameters, and radiation dose in both CM protocol groups

		Group 1 (Fixed-volume)	Group 2 (Body composition)	Mean difference	95 % CI	*p-* value
Total number of patients (n)		87	92			
Age (years)	Normal	59.3 ± 11.0 (34-76)	62.8 ± 9.3 (44-82)	−3.6	[−8.7, 1.6]	0.17
	Muscular	32.6 ± 11.8 (20-72)	34.0 ± 10.8 (19-56)	−1.4	[−7.5, 4.6]	0.63
	Overweight	58.5 ± 11.5 (31-75)	57.8 ± 14.4 (28-85)	0.7	[−6.0, 7.4]	0.84
Male (n) (%)	Normal	17 (57)	16 (50)	0.1	[−0.2, 0.3]	0.61
	Muscular	23 (85)	20 (69)	0.2	[−0.1, 0.4]	0.16
	Overweight	13 (43)	14 (45)	0.0	[−0.3, 0.2]	0.89
Total body weight (kg)	Normal	72.2 ± 9.7 (59-101)	67.8 ± 11.3 (38-88)	4.4	[−1.0, 9.8]	0.11
	Muscular	77.2 ± 12.5 (53-98)	73.6 ± 15.1 (54-118)	3.6	[−3.9, 11.1]	0.34
	Overweight	95.8 ± 16.3 (64-140)	93.4 ± 16.0 (70-136)	2.2	[−6.1, 10.5]	0.60
BMI (kg/m2)	Normal	23.4 ± 2.9 (19-33)	22.3 ± 2.8 (14-26)	1.2	[−0.3, 2.6]	0.11
	Muscular	23.6 ± 3.1 (19-33)	23.1 ± 3.2 (17-32)	0.4	[−1.3, 2.1]	0.61
	Overweight	31.6 ± 4.5 (25-46)	30.9 ± 3.7 (25-40)	0.7	[−1.4, 2.8]	0.51
Waist circumference (cm)	Normal	87.5 ± 8.2 (69-100)	84.5 ± 9.9 (64-100)	3.1	[−1.6, 7.7]	0.19
	Muscular	83.6 ± 10.2 (66-101)	82.0 ± 10.0 (66-102)	1.6	[−3.8, 7.0]	0.56
	Overweight	107.7 ± 10.5 (90-132)	107.9 ± 11.8 (88-131)	−0.2	[−5.9, 5.6]	0.95
CM volume (ml)	Normal	90.0	85.3 ± 14.9 (60-110)	4.7	[−0.7, 10.0]	0.09
	Muscular	90.0	106.2 ± 22.6 (80-170)	−16.2	[-24.9,–7.5]	<0.001
	Overweight	90.0	98.2 ± 16.0 (70-130)	−8.2	[-14.1,–2.4]	0.01
Flow rate (ml s^−1^)	Normal	5.0	4.7 ± 0.8 (3.3–6.0)	0.3	[−0.0, 0.6]	0.04
	Muscular	5.0	5.8 ± 1.0 (4.5–8.0)	−0.8	[-1.2,–0.4]	<0.001
	Overweight	5.0	5.4 ± 0.8 (4.0–7.2)	−0.4	[-0.7,–0.1]	0.01
CT dose index (mGy.cm)	Normal	6.4 ± 0.9 (4.4–9.5)	5.7 ± 1.0 (2.8–7.4)	0.6	[0.1, 1.1]	0.02
	Muscular	7.2 ± 1.1 (5.3–9.5)	5.9 ± 1.4 (3.8–9.6)	1.3	[0.6, 2.0]	<0.001
	Overweight	8.7 ± 1.4 (5.9–10.6)	8.2 ± 1.2 (6.0–10.2)	0.4	[−0.2, 1.1]	0.19
Body composition (n %)	Normal	30 (35)	32 (35)			
	Muscular	27 (31)	29 (32)			
	Overweight	30 (35)	31 (34)			

BMI, body mass index; CI, confidence interval; CM, contrast medium.

Note. Data are presented as mean ± standard deviation and ranges or percentages in parentheses.

Based on predefined factors and subjective assessment, the patients were divided into three body composition categories: (a), normal (*n* = 62); (b), muscular (*n* = 56); and (c), overweight (*n* = 61) (Table 2). The waist circumference was measured at the level of the umbilicus.^20,21^ Overweight were classified as BMI ≥25 and waist circumference ≥88 cm for females and ≥102 for males.^20,21^ A precondition for overweight categorisation was to fulfill requirements for both BMI and waist circumference. For the muscular categorisation, an age of >30 was set as initiation of age-related muscle loss.^22^ Therefore, all participants ≤30 years were included as muscular as long as none or only one of the predefined cut-offs for overweight was present.^20,21^ For patients ≥31 years, a subjective assessment was performed to determine if they belonged to the muscular group. The patient recruitment and categorisation were performed by a finite number of experienced CT radiographers (*n* = 18) trained in subjective assessment for this study.^23^

**Table 2.** Definitions of body composition categories according to age, body size factors and subjective assessment

	Normal	Muscular	Overweight
Total number of patients	62	56	61
18–30 (years)		29 (52%)	2 (3%)
> 31 (years)	62 (100%)	27 (48%)	59 (97%)
BMI (kg/m^2^)	<24.9	<24.9	≥25
Waist circumference (cm)	<88 cm (W)	<88 cm (W)	≥88 cm (W)
	<102 cm (M)	<102 cm (M)	≥102 cm (M)
Age (years)	>31	≤30 ≥31 when subjectively assessed as muscular	>18
Subjective assessment	No	Yes^*a*^	No

BMI, body mass index; M, men; W, women.

Note. In the normal and muscular category, only one of the predefined requirement for BMI and waist circumference was required. For the overweight category, both requirements for BMI and waist circumference were needed.

a Only when >30 years old.

### Scan protocol

Scans were performed using state-of-the-art CT scanner (Siemens SOMATOM Definition Force; Siemens Healthcare, Erlangen, Germany), with a 192 × 0.6 mm slice collimation, tube voltage 120 kV, reference tube current 130 mAs_ref_ (CareDose 4D^TM^, Siemens), pitch 2.5, and rotation time 0.25 s. Image reconstruction was performed with individually adapted field of view, 2.0 mm slice thickness, and increment of 1.5 mm. Image reconstruction used was Admire iterative reconstruction strength 2, br40 kernel (“std. soft”).

### CM parameters

A 16–20-gauge intravenous injection catheter was inserted in either left or right antecubital vein for CM administration. The CM concentration was 350 mgI ml^−1^ (Omnipaque; GE healthcare, Boston, MA), prewarmed to standardised 37^◦^C (99^◦^F) and injected with a power injector. The maximum pressure threshold was set to 325 psi for all injections.

The patients were randomised into two groups (Figure 1). Group 1 (*n* = 87) received a fixed CM volume of 90 ml, injected with a flow rate of 5 ml s^−1^. Group 2 (*n* = 92) received a body composition-tailored CM volume using a look-up table based on the patient weight and body composition (Table 3).

**Table 3.** Table for CM volume assessment using TBW and body composition-tailored strategy

TBW (kg)	Contrast volume (ml)		
	Normal	Muscular	Overweight
<50	60	70	50
51–55	65	80	55
56–60	70	85	60
61–65	80	90	65
66–70	85	100	70
71–80	95	110	80
81–90	110	125	90
91–100	120	140	100
101–110	130	155	110
111–120	145	170	120
>121	155	185	130

TBW, total body weight.

The injection duration was 18 s for both groups, consequently the flow rate was calculated individually for the patients in Group 2. CM injection was followed by a saline flush of 40 ml at the same flow rate. To minimise the artefacts caused by incoming CM in the thoracic veins, the image acquisition initiated 6 s after end of CM injection. The fixed scan delay was 24 s for both groups.

### Vascular attenuation and image quality

**Figure 2.**
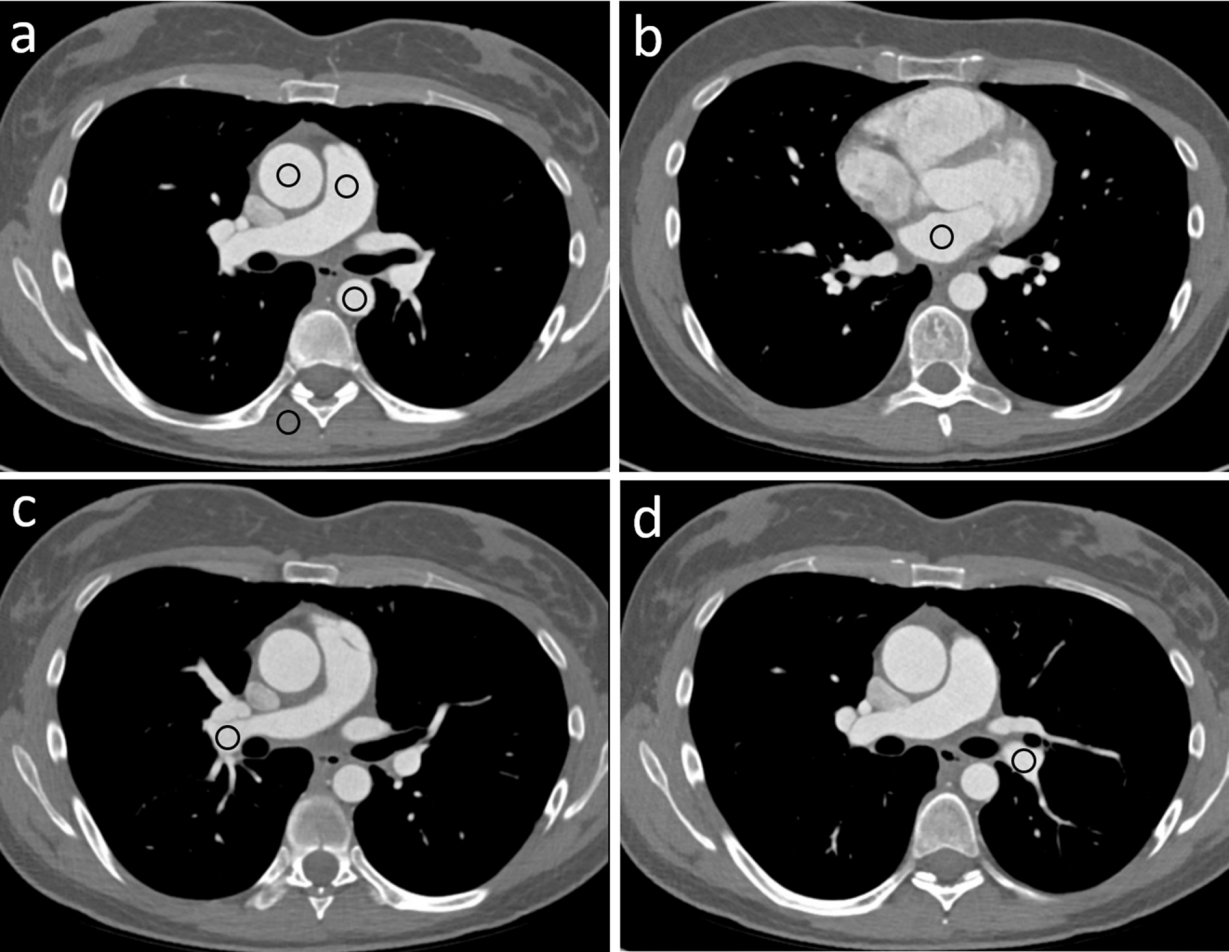
Measurements with ROIs in enhanced axial chest CT scans of the (**a**) pulmonary artery, ascending aorta, descending aorta, and paravertebral muscle; (**b**) left atrium; (**c**) right pulmonary artery; and (**d**) left pulmonary artery. ROI, region of interest.

For the primary outcome, CT number was measured using regions of interest (ROIs) in the pulmonary artery, ascending aorta and descending aorta, left atrium, right and left pulmonary artery, and the paravertebral muscle. ROIs were placed in the same anatomical levels between patients with approximate areas of 1.0 cm^2^ (Figure 2) or as large as the anatomic structure allowed in axial view. The intravascular attenuation was measured in Hounsfield units (HUs) with corresponding image noise (defined as the standard deviation (SD)). The overall mean intravascular attenuation was defined as the mean of the attenuation of all included thoracic vascular structures. Attenuation values of ≥250 HU were defined as diagnostic acceptable.^24,25^

Secondary, the paravertebral muscle measurements were used to calculate contrast-to-noise ratio (CNR) and signal-to-noise ratio (SNR). CNR and SNR were defined using the following equations^26^:

$$CNR=\frac{Mean\;intravascular\;attenuation\;\left(HU\right)-\;paravertebral\;muscle\;attenuation\;\left(HU\right)}{Paravertebral\;muscle\;attenuation\;\left(SD\right)}$$

$$SNR=\;\frac{Mean\;intravascular\;attentuation\;\left(HU\right)}{Mean\;intravascular\;attenuation\;\left(SD\right)}$$

### Statistical analysis

Continuous data were presented as the mean ± SD. Absolute numbers and percentages were used for categorical variables. Differences between groups were analysed using Student’s *t*-test, while variations in attenuation between body composition categories were compared for both groups using one-way ANOVA followed by *post hoc* Tuckey test to compare differences between each body composition category. Pearson’s χ^2^ or Fisher’s exact test was used as appropriate to calculate differences between categorical variables. Correlation of data was assessed using Pearson’s correlation coefficient. Data analysis was performed with STATA v. 16.0 (StataCorp LLX, College Station, TX). Statistical significance was accepted at the 0.05 level.

## Results

### Patient characteristics

The demographic characteristics of the study population are summarised in Table 1. No statistical differences in demographics characteristics were found between or within the two included groups.

### Injection parameters

**Figure 3.**
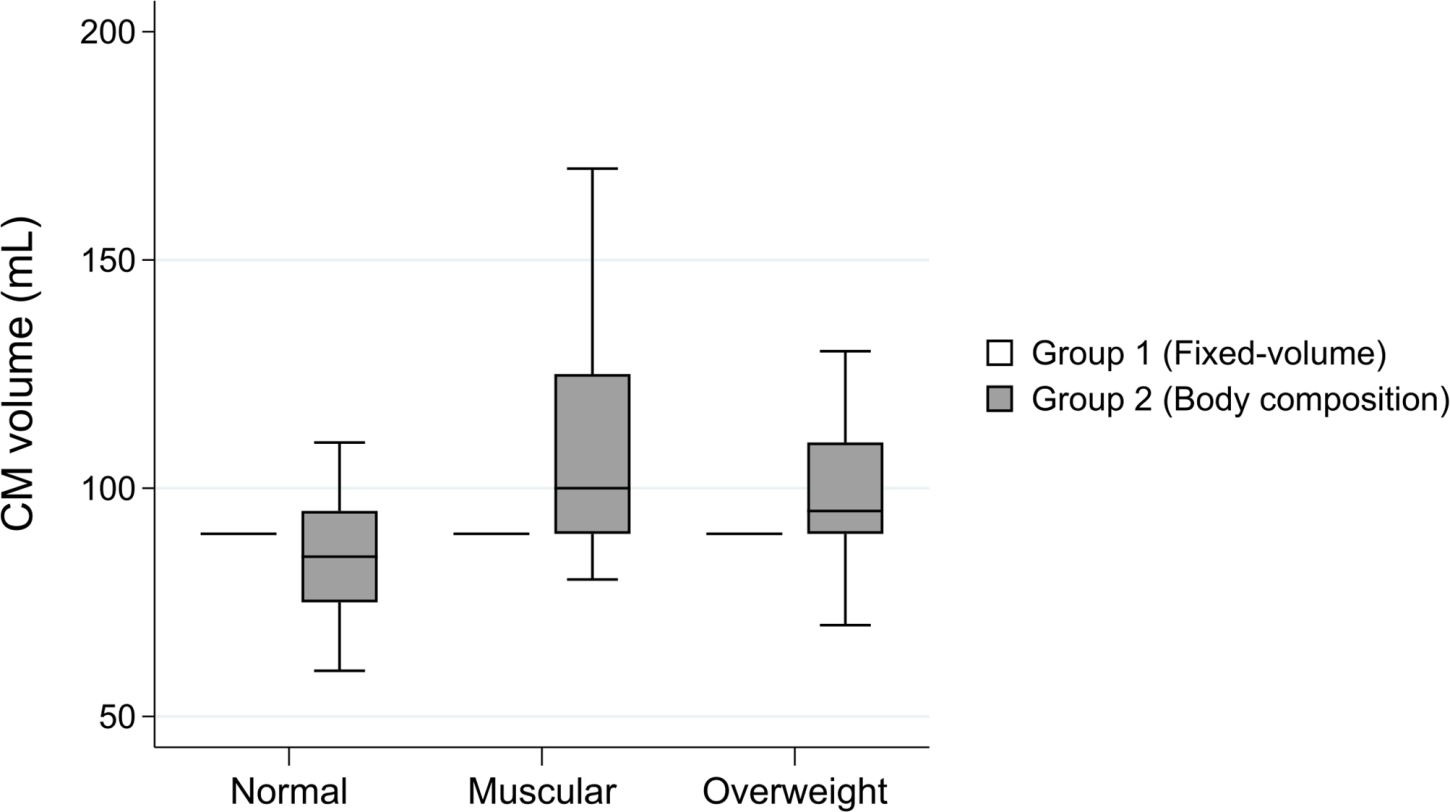
Box and whiskers plot of the CM volume (ml) for both CM protocols and all three included body composition categories. Note. The single lines without surrounding box plot illustrates Group 1 receiving a fixed CM volume. CM, contrast medium.

The comparisons of CM parameters between the two groups are shown in Table 1. There were significant differences between the groups for CM volume and flow rate when comparing both the muscular and overweight categories with each other. No statistical differences in CM volume was seen between the groups for patients with normal body composition, however, flow rate was significantly different (*p* = 0.043). Figure 3 demonstrates the variation in CM volume for the two CM protocols used in this study. The Tukey test for the different body composition categories in Group 2 showed a significant difference in CM volume for the normal (mean 85.3 ml) *vs* the muscular (mean 106.2 ml; *p* = <0.001) and overweight (mean 98.2 ml; *p* = 0.01) body composition categories.

### Vascular attenuation and image quality

In Group 1, the overall mean vascular attenuation values for each body composition category (normal, muscular, overweight) were 385 ± 76 HU, 346 ± 59 HU, and 342 ± 71 HU, respectively (Table 4). A significant difference in mean attenuation was found between the normal and overweight category (*p* = 0.047), but not between the other categories. In Group 2, the overall mean attenuation values were 374 ± 81 HU, 396 ± 63 HU, and 367 ± 57 HU for the normal, muscular and overweight categories, respectively. There were no significant differences between the categories.

**Table 4.** Image quality assessment of thoracic structures using fixed-volume CM protocol (Group 1) and body composition CM protocol (Group 2)

		Vascular attenuation (HU)				Image noise (HU)				CNR			
		Group 1 Mean ± SD	Group 2 Mean ± SD	Mean difference	*p-* value	Group 1 Mean ± SD	Group 2 Mean ± SD	Mean difference	*p-* value	Group 1 Mean ± SD	Group 2 Mean ± SD	Mean difference	*p-* value
Overall CM structures	Normal	385 ± 76	374 ± 81	11	0.61	18 ± 2	17 ± 2	1	0.44	18 ± 6	20 ± 6	2	0.33
	Muscular	346 ± 59	396 ± 63	50	0.004	15 ± 2	17 ± 2	2	<0.001	19 ± 7	20 ± 7	1	0.67
	Overweight	342 ± 71	367 ± 57	25	0.12	19 ± 2	19 ± 2	0	0.28	16 ± 5	16 ± 5	0	0.62
Pulmonary trunc	Normal	376 ± 104	366 ± 122	10	0.71	16 ± 4	17 ± 4	1	0.82	18 ± 6	19 ± 7	1	0.46
	Muscular	352 ± 72	364 ± 88	12	0.58	13 ± 2	15 ± 3	2	0.009	20 ± 7	18 ± 7	2	0.38
	Overweight	335 ± 92	357 ± 89	22	0.35	18 ± 4	19 ± 4	1	0.63	15 ± 6	16 ± 6	1	0.84
Right pulmonary artery	Normal	386 ± 100	398 ± 131	12	0.69	18 ± 4	18 ± 4	0	0.72	18 ± 6	21 ± 8	3	0.12
	Muscular	354 ± 77	379 ± 95	25	0.28	18 ± 4	19 ± 4	1	0.06	20 ± 7	19 ± 8	1	0.70
	Overweight	346 ± 99	378 ± 90	32	0.18	18 ± 4	20 ± 3	2	0.06	16 ± 6	17 ± 6	1	0.56
Left pulmonary artery	Normal	394 ± 102	399 ± 127	5	0.86	21 ± 5	21 ± 5	0	0.83	19 ± 6	21 ± 8	2	0.19
	Muscular	358 ± 76	385 ± 91	27	0.24	19 ± 4	20 ± 4	1	0.26	20 ± 7	19 ± 7	1	0.68
	Overweight	343 ± 95	384 ± 96	41	0.10	20 ± 4	22 ± 4	2	0.26	16 ± 7	17 ± 7	1	0.43
Left atrium	Normal	382 ± 63	364 ± 68	18	0.27	20 ± 3	18 ± 3	2	0.04	18 ± 5	19 ± 6	1	0.49
	Muscular	333 ± 61	402 ± 55	69	<0.001	17 ± 3	19 ± 3	2	0.02	19 ± 7	20 ± 7	1	0.25
	Overweight	333 ± 67	354 ± 45	21	0.17	21 ± 3	21 ± 3	0	0.79	15 ± 5	16 ± 4	1	0.71
Aorta ascendens	Normal	393 ± 68	367 ± 64	26	0.12	15 ± 3	14 ± 2	1	0.30	18 ± 6	19 ± 5	1	0.80
	Muscular	345 ± 65	424 ± 57	79	<0.001	13 ± 3	14 ± 2	1	0.012	19 ± 7	22 ± 7	3	0.19
	Overweight	352 ± 70	371 ± 48	19	0.21	17 ± 3	17 ± 3	0	0.64	16 ± 5	16 ± 4	0	0.76
Aorta descendens	Normal	376 ± 76	353 ± 60	23	0.19	15 ± 2	15 ± 2	0	0.24	18 ± 6	18 ± 5	0	0.82
	Muscular	337 ± 66	424 ± 58	87	<0.001	13 ± 2	15 ± 2	2	0.005	18 ± 7	22 ± 7	4	0.12
	Overweight	341 ± 73	362 ± 45	21	0.21	16 ± 3	17 ± 3	1	0.72	16 ± 6	16 ± 5	0	0.73

CM, contrast medium; CNR, contrast-to-noise ratio; HU, hounsfield units; SD, standard deviation.

**Figure 4.**
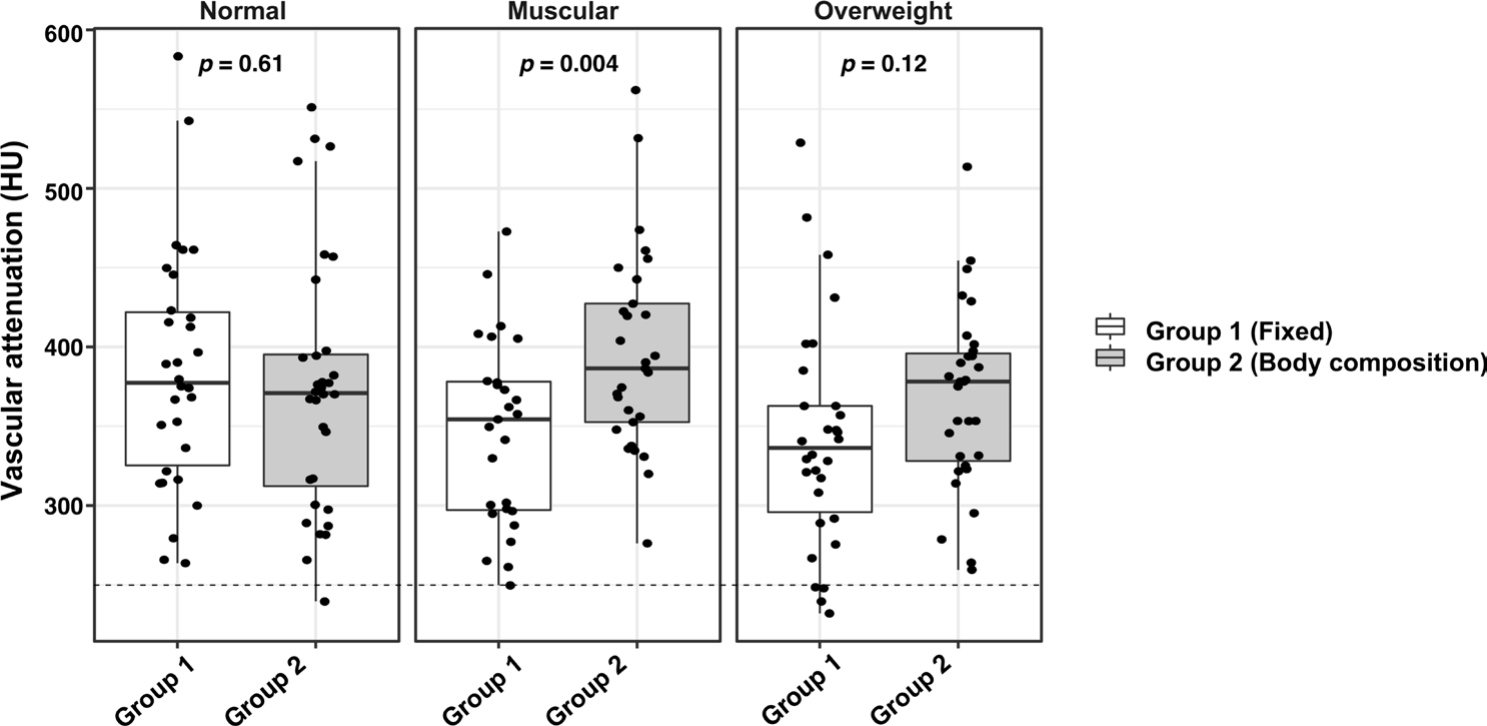
Box and whisker plot for both contrast medium protocols and all body composition categories. The horizontal dotted reference line indicates an empirical diagnostic attenuation threshold of 250 HU.

In average, Group 1 showed markedly lower overall attenuation values in the muscular and overweight categories compared to Group 2 (*p* = 0.004 and *p* = 0.12, respectively), while the normal category patients showed increased attenuation (*p* = 0.61) (Figure 4). A significant difference in mean attenuation values was demonstrated between the two CM groups in left atrium, ascending aorta, and descending aorta for the muscular body composition category (all *p* = <0.001) (Table 4).

Mean diagnostic attenuation of ≥250 HU was reached for all body categories for overall mean intravascular attenuation, and separately for all included vascular structures, regardless of CM protocol (Table 4). However, 13% (4/31) of the patients in the overweight category receiving a fixed CM volume of 90 ml did not reach the desired attenuation level (250 HU) (Figure 4). This difference in number of outliers between the two groups was close to significant (*p* = 0.053). In Group 2, one patient in the normal body composition category measured below the preferred attenuation level. No significant difference in attenuation was found between patients <30 years old when compared to patients >30 years for Group 1 and Group 2 (*p* = 0.81 and *p* = 0.11, respectively).

Tables 3 and 4 show CT dose index_vol_ and noise. A statistical difference in mean image noise (HU) between the two groups was observed for the muscular category for overall CM structures (*p* = <0.001) and pulmonary trunc (*p* = 0.01). No significant differences in CNR was found between the two groups (Table 4). Calculations of SNR also showed no significant differences, thus confirmed comparable image quality (data not shown).

### Correlation values

**Figure 5.**
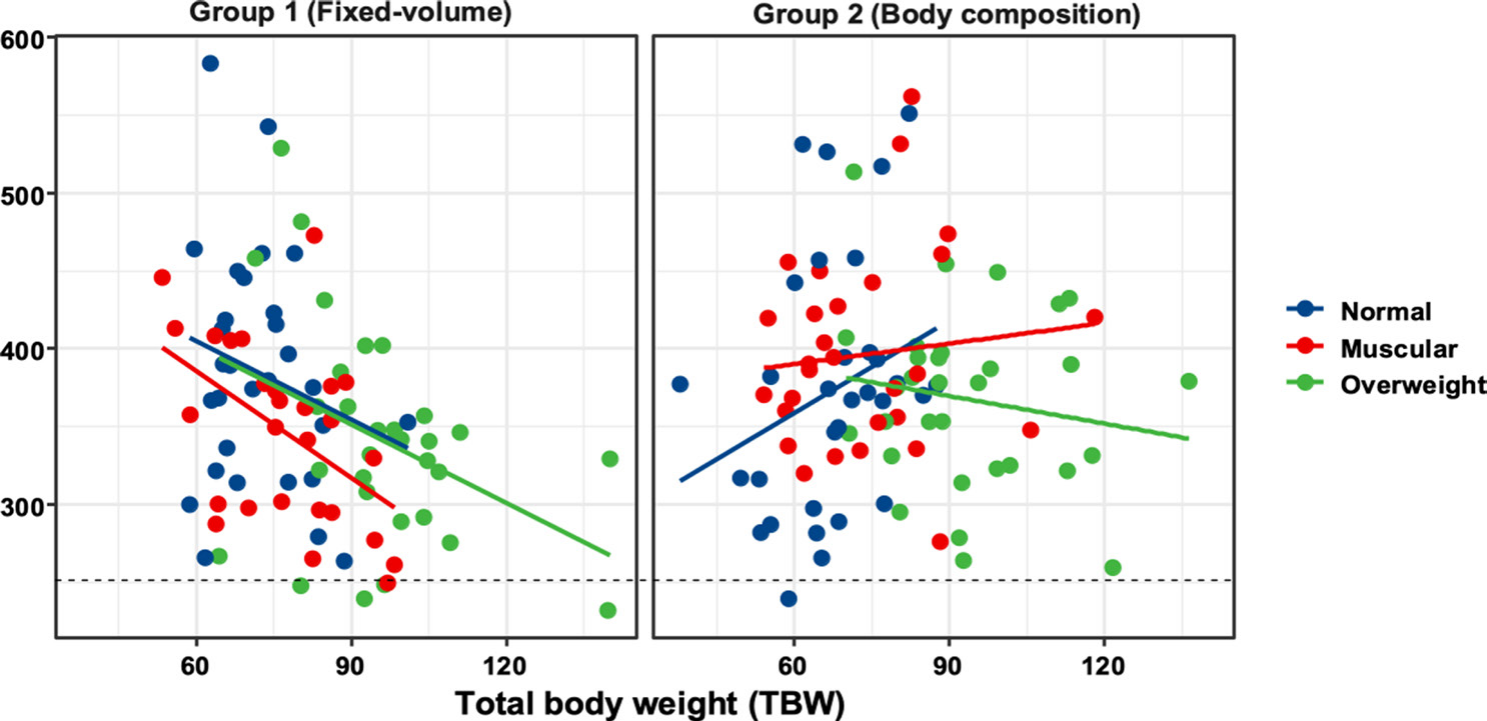
Plot of vascular attenuation (HU) with regard to TBW. The horizontal dotted reference line indicates an empirical diagnostic attenuation threshold of 250 HU. The trend lines demonstrate an inverse correlation between vascular attenuation and TBW using fixed contrast medium protocol for all body composition groups. HU, Hounsfield unit; TBW, total body weight

Figure 5 illustrates the correlations between mean vascular attenuation and TBW for all body composition categories in both CM protocol groups.

In Group 1, moderate but significant negative correlations were found in the muscular category between CM attenuation and TBW (*r* = −0.49; *p* = 0.009), gender (*r* = −0.52; *p* = 0.005), BMI (*r* = −0.44; *p* = 0.02), and waist circumference (*r* = −0.39; *p* = 0.04). In the overweight category, moderate but significant negative correlation was also noted between CM attenuation and TBW (*r* = −0.39; *p* = 0.03), and waist circumference (*r* = −0.50; *p* = 0.005). In the normal category, there were no significant results, but negative correlation was noted between attenuation and TBW (*r* = −0.22; *p* = 0.25).

In Group 2, there were no significant correlations between CM attenuation and TBW. In the muscular category moderate but significant positive correlation was found between attenuation and age (*r* = 0.51; *p* = 0.005), indicating increased vascular enhancement with age. In the normal body category, moderate but significant positive correlations were noted between attenuation and BMI (*r* = 0.39; *p* = 0.03), and waist circumference (*r* = .46; *p* = 0.008). A low positive correlation was also found in the muscular group for the same demographic factors, whereas weak negative correlations were noted for the overweight group, none being significant.

## Discussion

Using a weight and body composition-tailored CM protocol in chest CT resulted in comparable enhancement between three different body composition categories. No significant differences in attenuation were found. In contrast, with the fixed-volume protocol, higher attenuation levels were observed in patients with normal body composition than for patients with muscular and overweight body composition, indicating suboptimal use of CM.

As expected, only minor differences between the groups in CM parameters were noted for the normal body composition category, resulting in comparable vascular attenuation. However, for the muscular and overweight body composition category, CM parameters (volume and flow rate) were significantly different, with a higher CM volume and flow rate utilised in Group 2. For the muscular category, this increase of mean 16 ml and 0.8 ml s^−1^ led to a significant raise in vascular attenuation. Furthermore, in Group 2, there was less variability in attenuation than in Group 1 for muscular and overweight body composition patients. Therefore, our results indicate a more homogenous enhancement and fewer outliers when CM volume is personalised. In Group 2, both CM volume and injection rate are tailored to each patient weight and body composition, thus with this approach, the injection rate per kilogram of body weight is the same. Therefore, the optimal timing for vascular enhancement should be almost constant. Some studies have investigated this approach in abdominal CT.^27,28^

There is a lack of studies investigating the impact of patient-related factors on CM enhancement in chest CT. However, several authors have investigated the use of TBW with more personalised strategies within pulmonary, cardiac and abdominal CT.^5,10,16,17,29,30^ Given the global rise in overweight and obesity, approaches that stratify CM doses across a range of body compositions are favored. Ratnakanthan et al^5^ investigated the variation in pulmonary artery enhancement between an individual adapted CM protocol using TBW and a fixed strategy in pulmonary CT angiography. A more homogenous enhancement was shown when individually tailored CM volume was used, especially in obese patients.^5^ These findings are in line with our results; fixed CM protocol caused significantly lower mean vascular attenuation in the overweight body composition category than in the normal composition category. However, as expected, the slope of the fitted regression line was similar for normal and overweight composition patients due to the relatively smaller blood volume per TBW in overweight patients. In other words, the same decrease in CM enhancement occurred with increased TBW in both overweight and normal composition patients. When adjusting CM volume to TBW and body composition, the association between CM enhancement and TBW was still negative for overweight patients in Group 2, however, to a lesser extent than for overweight patients in Group 1. This may indicate a more limited dispersing effect from not metabolically active body fat in the early CM phase. Notably, the CM volume was lowest per kg TBW for this body composition category. As a result, the highest number of patients below the diagnostic acceptance value of ≥250 HU was found in this body composition category using fixed-volume. Although the difference was not significant between the two groups of overweight patients, the lower attenuation levels may be clinically relevant. In addition, the steeper negative regression slope using fixed CM volume in muscular patients may indicate a higher diluting effect in this category.

In Group 1, there was a negative correlation between thoracic vascular attenuation and both TBW and waist circumference for all body categories. This indicates that an increase of body size leads to decrease in enhancement levels. In comparison, no significant correlation was found in Group 2 for the same body composition parameters for muscular and overweight categories indicating a more consistent enhancement level across various body size parameters, thus reduced under- and overdosage of CM.

As routine chest CT is utilised for a wide range of clinical conditions, the preferred scan delay and scan timing is highly dependent on the clinical indications and diagnostic target organs.^31^ In the current study, a significant difference in vascular enhancement was observed in ascending aorta, descending aorta, and left atrium between the two strategies (all *p* = <0.001) for muscular body composition patients. This indicates a more pronounced diluting effect in the systemic circulation, compared to the pulmonary circulation. As a longer delay may be preferential for parenchymal and venous enhancement,^32,33^ the prolonged effect of diluting the CM in the blood for various body compositions, especially in muscular patients, may be more prominent. Our results indicate that individualisation to TBW and body composition may be of greater importance in delayed phases than earlier phases of chest CT. However, higher volumes may be considered for younger and/or muscular patients.^34^

In this study, the CNR and SNR were not significantly different between the two groups supporting the use of a body composition-tailored protocol. These findings are consistent with previous published studies.^8,17^

Although mainly objective measures was utilised in the current study for body composition assessment, this modified look-up table also facilitates a coarser division of body composition by using only TBW together with observable muscle and body fat mass. Consequently, this approach can maintain clinical efficiency, reduce interpatient variability, and be easy applicable in bedridden patients.

This study has several limitations. First, the scan protocols did not take into account variations in cardiac output, which may introduce some variability. Second, the inclusion of patients for the muscular body composition categories partly included subjective assessment. Although a bioelectric impedance analyser, calculating fat-free mass may have been more accurate, a limited number of trained radiographers performed the assessment.^23^ Third, the age limit introduce some uncertainties with regard to age-related muscle loss. Although aging affects a wide range of physiological processes, the most observable are reported being body composition changes including loss of muscle mass and increased body fat.^35,36^ Moreover, no significant difference in enhancement was found in the muscular category for patients under or over 30 years old. Also, the study does not include subjective image quality assessment. Although these analyses are important, other studies have consistently reported corresponding results between objective CM enhancement and subjective image quality assessment.^7,8,14^ Lastly, the sample size could be considered relatively small.

In conclusion, a fixed-volume CM protocol in chest CT showed large variations between different body compositions indicating suboptimal use of CM. In comparison, a combined TBW and body composition-tailored CM protocol resulted in more homogenous enhancement for all body composition categories with fewer outliers.

## References

[1] Bae KT . Intravenous contrast medium administration and scan timing at CT: considerations and approaches. Radiology 2010; 256: 32–61. doi: 10.1148/radiol.10090908 20574084

[2] Henning MK, Aaløkken TM, Johansen S . Contrast medium protocols in routine chest CT: a survey study. Acta Radiol 2022; 63: 351–59. doi: 10.1177/0284185121997111 33648351

[3] Caruso D, Rosati E, Panvini N, Rengo M, Bellini D, Moltoni G, et al . Optimization of contrast medium volume for abdominal CT in oncologic patients: prospective comparison between fixed and lean body weight-adapted dosing protocols. Insights Imaging 2021; 12(1): 40. doi: 10.1186/s13244-021-00980-0 33743100 PMC7981367

[4] Hendriks BMF, Kok M, Mihl C, Bekkers SCAM, Wildberger JE, Das M . Individually tailored contrast Enhancement in CT pulmonary angiography. Br J Radiol 2016; 89(1061): 20150850. doi: 10.1259/bjr.20150850 26689096 PMC4985462

[5] Ratnakanthan PJ, Kavnoudias H, Paul E, Clements WJ . Weight-adjusted contrast administration in the computed tomography evaluation of pulmonary embolism. J Med Imaging Radiat Sci 2020; 51: 451–61. doi: 10.1016/j.jmir.2020.06.002 32620525

[6] George AJ, Manghat NE, Hamilton MCK . Comparison between a fixed-dose contrast protocol and a weight-based contrast dosing protocol in abdominal CT. Clin Radiol 2016; 71: 1314. doi: 10.1016/j.crad.2016.07.009 27557991

[7] Mihl C, Kok M, Altintas S, Kietselaer B, Turek J, Wildberger JE, et al . Evaluation of individually body weight adapted contrast media injection in coronary CT-angiography. Eur J Radiol 2016; 85: 830–36. doi: 10.1016/j.ejrad.2015.12.031 26971431

[8] Martens B, Hendriks BMF, Eijsvoogel NG, Wildberger JE, Mihl C . Individually body weight-adapted contrast media application in computed tomography imaging of the liver at 90 kVp. Invest Radiol 2019; 54: 177–82. doi: 10.1097/RLI.0000000000000525 30721159

[9] Zanardo M, Doniselli FM, Esseridou A, Tritella S, Mattiuz C, Menicagli L, et al . Abdominal CT: a Radiologist-driven adjustment of the dose of iodinated contrast agent approaches a calculation per lean body weight. Eur Radiol Exp 2018; 2(1): 41. doi: 10.1186/s41747-018-0074-1 30515613 PMC6279751

[10] Peet K, Clarke SE, Costa AF . Hepatic Enhancement differences when dosing iodinated contrast media according to total versus lean body weight. Acta Radiol 2019; 60: 807–14. doi: 10.1177/0284185118801137 30227724

[11] Masuda T, Nakaura T, Funama Y, Sato T, Higaki T, Kiguchi M, et al . Effect of patient characteristics on vessel Enhancement at lower extremity CT angiography. Korean J Radiol 2018; 19: 265–71. doi: 10.3348/kjr.2018.19.2.265 29520184 PMC5840055

[12] Davenport MS, Parikh KR, Mayo-Smith WW, Israel GM, Brown RKJ, Ellis JH . Effect of fixed-volume and weight-based dosing regimens on the cost and volume of administered iodinated contrast material at abdominal CT. J Am Coll Radiol 2017; 14: 359–70. doi: 10.1016/j.jacr.2016.09.001 28017270

[13] Benbow M, Bull RK . Simple weight-based contrast dosing for standardization of portal phase CT liver Enhancement. Clin Radiol 2011; 66: 940–44. doi: 10.1016/j.crad.2010.12.022 21724182

[14] Perrin E, Jackson M, Grant R, Lloyd C, Chinaka F, Goh V . Weight-adapted iodinated contrast media administration in Abdomino-pelvic CT: can image quality be maintained? Radiography (Lond) 2018; 24: 22–27. doi: 10.1016/j.radi.2017.08.011 29306370

[15] Walgraeve M-S, Pyfferoen L, Van De Moortele K, Zanca F, Bielen D, Casselman JW . Implementation of patient-tailored contrast volumes based on body surface area and heart rate Harmonizes contrast Enhancement and reduces contrast load in small patients in portal venous phase abdominal CT. Eur J Radiol 2019; 121: 108630. doi: 10.1016/j.ejrad.2019.07.031 31587920

[16] Costa AF, Peet K, Abdolell M . Dosing iodinated contrast media according to lean versus total body weight at abdominal CT: A stratified randomized controlled trial. Acad Radiol 2020; 27: 833–40. doi: 10.1016/j.acra.2019.07.014 31439467

[17] Eijsvoogel NG, Hendriks BMF, Nelemans P, Mihl C, Willigers J, Martens B, et al . Personalization of CM injection protocols in coronary computed Tomographic angiography (people CT trial). Contrast Media Mol Imaging 2020; 2020: 5407936. doi: 10.1155/2020/5407936 32410922 PMC7201621

[18] Collaborators G, Afshin A, Forouzanfar MH . Health effects of overweight and obesity in 195 countries over 25 years. N Engl J Med 2017; 377: 13–27. doi: 10.1056/NEJMoa1614362 28604169 PMC5477817

[19] Henning MK, Gunn C, Arenas-Jiménez J, Johansen S . Strategies for calculating contrast media dose for chest CT. Eur Radiol Exp 2023; 7: 29. doi: 10.1186/s41747-023-00345-w 37303003 PMC10258182

[20] Obesity: preventing and managing the global epidemic. World Health Organ Tech Rep Ser 2000; 894: 1–253. 11234459

[21] Clinical guidelines on the identification, evaluation, and treatment of overweight and obesity in adults--the evidence report. National Institutes of Health Obesity Research 1998; 6: 51S–209S. 9813653

[22] Mitchell WK, Williams J, Atherton P, Larvin M, Lund J, Narici M . Sarcopenia, Dynapenia, and the impact of advancing age on human Skeletal muscle size and strength; a quantitative review. Front Physiol 2012; 3: 260. doi: 10.3389/fphys.2012.00260 22934016 PMC3429036

[23] Dastan K, Henning MK, England A, Aalokken TM, Johansen S . An investigation into the variability of Radiographers assessing body composition prior to CT contrast media administration. Radiography (Lond) 2021; 27: 168–72. doi: 10.1016/j.radi.2020.07.012 32855023

[24] Bae KT, Tao C, Gürel S, Hong C, Zhu F, Gebke TA, et al . Effect of patient weight and scanning duration on contrast Enhancement during pulmonary Multidetector CT angiography. Radiology 2007; 242: 582–89. doi: 10.1148/radiol.2422052132 17255426

[25] Haidary A, Bis K, Vrachliotis T, Kosuri R, Balasubramaniam M . Enhancement performance of a 64-slice triple rule-out protocol vs 16-slice and 10-slice Multidetector CT-angiography protocols for evaluation of aortic and pulmonary vasculature. J Comput Assist Tomogr 2007; 31: 917–23. doi: 10.1097/rct.0b013e318040aded 18043357

[26] Jensen K, Andersen HK, Smedby Ö, Østerås BH, Aarsnes A, Tingberg A, et al . Quantitative measurements versus receiver operating characteristics and visual grading regression in CT images reconstructed with Iterative reconstruction: A phantom study. Acad Radiol 2018; 25: 509–18. doi: 10.1016/j.acra.2017.10.020 29198945

[27] Awai K, Hiraishi K, Hori S . Effect of contrast material injection duration and rate on aortic peak time and peak Enhancement at dynamic CT involving injection protocol with dose tailored to patient weight. Radiology 2004; 230: 142–50. doi: 10.1148/radiol.2301021008 14695390

[28] Costa AF, Peet K . Contrast media injection protocol for Portovenous phase abdominal CT: does a fixed injection duration improve hepatic Enhancement over a fixed injection rate? Abdom Radiol (NY) Abdom Radiol 2021; 46: 2968–75. doi: 10.1007/s00261-020-02919-3 33386915

[29] Bae KT, Seeck BA, Hildebolt CF, Tao C, Zhu F, Kanematsu M, et al . Contrast Enhancement in cardiovascular MDCT: effect of body weight, height, body surface area, body mass index, and obesity. AJR Am J Roentgenol 2008; 190: 777–84. doi: 10.2214/AJR.07.2765 18287452

[30] Yamashita Y, Komohara Y, Takahashi M, Uchida M, Hayabuchi N, Shimizu T, et al . Abdominal Helical CT: evaluation of optimal doses of intravenous contrast material--a prospective randomized study. Radiology 2000; 216: 718–23. doi: 10.1148/radiology.216.3.r00se26718 10966700

[31] Bae KT . Optimization of contrast Enhancement in Thoracic MDCT. Radiol Clin North Am 2010; 48: 9–29. doi: 10.1016/j.rcl.2009.08.012 19995627

[32] Best TD, Mercaldo SF, Bryan DS, Marquardt JP, Wrobel MM, Bridge CP, et al . Multilevel body composition analysis on chest computed tomography predicts hospital length of stay and complications after Lobectomy for lung cancer: A multicenter study. Ann Surg 2022; 275: e708–15. doi: 10.1097/SLA.0000000000004040 32773626

[33] Sundaram B, Kuriakose JW, Stojanovska J, Watcharotone K, Parker RA, Kazerooni EA . Thoracic central venous evaluation: comparison of first-pass direct versus delayed-phase indirect Multidetector CT Venography. Clin Imaging 2015; 39: 412–16. doi: 10.1016/j.clinimag.2015.02.005 25724223 PMC4412308

[34] Itoh S, Ikeda M, Satake H, Ota T, Ishigaki T . The effect of patient age on contrast Enhancement during CT of the Pancreatobiliary region. AJR Am J Roentgenol 2006; 187: 505–10. doi: 10.2214/AJR.05.0541 16861556

[35] JafariNasabian P, Inglis JE, Reilly W, Kelly OJ, Ilich JZ . Aging human body: changes in bone, muscle and body fat with consequent changes in nutrient intake. J Endocrinol 2017; 234: R37–51. doi: 10.1530/JOE-16-0603 28442508

[36] Hunter GR, Gower BA, Kane BL . Age related shift in visceral fat. Int J Body Compos Res 2010; 8: 103–8. 24834015 PMC4018766

